# Transient Damped Response of a 3D-Printed Composite Cantilever Beam

**DOI:** 10.3390/ma19143074

**Published:** 2026-07-16

**Authors:** Miroslaw Wesolowski, Naftal Kaleb Ngughu, Jon Aurrekoetxea Narbarte

**Affiliations:** 1 Faculty of Civil Engineering, Environmental and Geodetic Sciences, Koszalin University of Technology, 75-453 Koszalin, Poland; naftal.ngughu@s.tu.koszalin.pl; 2 Mechanical and Industrial Production Department, Mondragon Unibertsitatea, 20500 Mondragón, Spain

**Keywords:** 3D printing, composite beam, transient analysis, modal damping, finite element analysis, CLPT

## Abstract

This study presents a transient dynamic analysis of a 3D-printed composite cantilever beam fabricated from short carbon fibre-reinforced polyamide (CF-PA-12). Particular attention is given to acceleration response, vibration damping, and energy dissipation, which govern the transient behaviour and dynamic stability of lightweight composite structures under impulsive loading. The research combines experimental modal analysis (EMA) and transient impact testing with numerical simulations based on classical laminated plate theory (CLPT). A finite element model was developed in Simulia/Abaqus and used within a modal-superposition-based transient framework incorporating experimentally identified damping ratios and measured impact forces. The proposed approach enables realistic prediction of vibration decay and time-dependent acceleration response. Good agreement between experimental and numerical results confirms the capability of the method to reproduce the dynamic behaviour of additively manufactured composite beams subjected to impact excitation.

## 1. Introduction

Additive manufacturing (AM) has evolved beyond its initial role as a prototyping technique and is now widely used for low-volume production, allowing components to be fabricated for immediate application [1]. Unlike conventional manufacturing methods, it enables the fabrication of complex geometries, including lattice [2] and functionally graded structures [3], that were previously impossible or economically unfeasible. Among AM techniques, fused deposition modelling (FDM) has gained attention for its ability to produce composite structures reinforced with short [4,5] or continuous fibres [6,7]. The combination of polymer matrices with reinforcing fibres enables the design of materials with tailored stiffness, strength, and damping properties. Nevertheless, the technological readiness of FDM composite structures for certified load-bearing applications remains limited, particularly where reliable prediction of dynamic response is required. This limitation arises partly from the absence of comprehensive structural design and qualification guidelines for FDM composite components, especially for forced dynamic responses under transient and steady-state loading. Addressing these challenges through combined experimental and numerical investigations is essential to enable the reliable design and qualification of additively manufactured polymer composites as load-bearing structural members across a wide range of industrial sectors.

Transient analysis is essential for structures subjected to time-dependent dynamic loading. Unlike steady-state or purely modal analyses, transient analysis enables the prediction of the structural response under short-duration, impulsive, or rapidly varying excitations, which are commonly encountered in practical engineering systems. Accurate prediction of transient behaviour is crucial in this context, as dynamic loads may induce significant vibrations that influence structural integrity, fatigue life, and aeroelastic stability. Transient and forced vibration analyses are widely used in aerospace engineering. Structural components such as aircraft wings [8,9] and helicopter rotor blades [10,11] are often idealised as cantilever plates or beams to simplify their dynamic modelling while preserving the essential bending and torsional behaviour. Such simplified models are often used to study the response of these structures to aerodynamic loads, gust excitations, impact events, and control-induced forces. Similarly, civil engineering structures frequently require transient dynamic analysis to evaluate their behaviour under environmental and operational loads. Many slender structures, including communication towers [12], masts [13], industrial chimneys [14], and wind turbine towers [15], can be effectively approximated as cantilever beam systems when analysing their dynamic response. These structures are exposed to transient wind loads, seismic excitations, start-up operations, and accidental loads, all of which generate time-varying structural responses.

The increasing industrial use of additively manufactured composite structures requires reliable prediction of their static and dynamic response. Since composite structures in the form of beams and plates are among the most commonly used structural elements, it is natural to devote the majority of attention to these types of structures. In particular, understanding how these structures behave under transient vibrations is important for evaluating their safety, serviceability, and fatigue performance. One of the main challenges in analysing such systems is that the applied load is typically obtained from experimental data, which makes it necessary to rely on numerical methods to determine the vibration response. Two main approaches are commonly used to compute transient structural responses: direct time integration and frequency-domain methods based on modal superposition. Direct integration techniques, such as the Newmark method [16,17,18], solve the full system in a step-by-step fashion in time. This makes them well suited for capturing nonlinear behaviour and high-frequency effects, but they can also become computationally expensive, particularly for large models or parametric studies. Similarly, the modal superposition method is widely used to analyse the transient response of cantilever beams and plates subjected to impulsive loading [19]. This formulation is especially effective for linear or lightly damped systems, as it allows the dynamic response to be computed efficiently while also providing clear insight into how different mode shapes contribute under various loading conditions. Previous studies have shown that, for beam and plate structures, a limited number of mode shapes can provide reasonable results for transient response analysis [20]. Finite element studies further confirm that modal superposition is an effective approach for computing impulse responses once the eigenvalue problem has been solved [21]. The method has also been successfully applied to composite and functionally graded structures, forming the basis for both analytical and numerical predictions of transient response [22]. Transient behaviour of dynamically loaded structures is strongly influenced by vibration damping and energy dissipation mechanisms. In composite structures, this dissipation process is particularly complex because it originates from multiple sources, including matrix viscoelasticity, fibre-matrix interactions, interlayer friction, and microstructural defects introduced during manufacturing. For additively manufactured polymer composites, the layered nature of the fabrication process further increases the importance of accurately characterising damping behaviour, since interlayer bonding quality, filament orientation, and process-induced porosity can strongly influence vibration attenuation and transient response characteristics [23,24].

Accurate representation of damping is therefore essential when predicting the transient response of lightweight composite structures. Inaccuracies in damping estimation may lead to discrepancies in vibration amplitudes, decay rates, and energy dissipation. A further advantage of the superposition method is that it is a linear procedure, which makes it straightforward to include modal damping ratios obtained either from experimental modal analysis [25] or from analytical approaches, such as energy-based methods [26].

In addition to accurate damping characterisation, reliable constitutive modelling is essential for the industrial implementation of 3D-printed composite structures. Inaccurate representation of material behaviour may lead to significant errors in predicted stiffness, strength, and fatigue life, ultimately resulting in unsafe or overly conservative designs. Various modelling strategies have therefore been proposed to describe the mechanical behaviour of FDM composites. At the macroscale, finite element analyses commonly employ homogenised [27,28] or equivalent material properties [29], whereas mesoscale approaches explicitly represent individual filaments or layers to account for interlayer interactions [30]. Since each deposited filament layer can be regarded as an orthotropic lamina, the mechanical behaviour of FDM structures closely resembles that of conventional laminated composites. Consequently, classical laminated plate theory (CLPT), which has been successfully applied to the static analysis of FDM structures [31], represents a promising framework for modelling their structural response. However, its application to the prediction of transient dynamic behaviour of additively manufactured composite structures has received only limited attention and remains insufficiently validated experimentally.

Despite the considerable progress achieved in the dynamic analysis of composite structures, several important challenges remain unresolved for additively manufactured FDM composites. Existing studies have primarily focused on modal characteristics, free vibration behaviour, or static mechanical performance, whereas comparatively little attention has been devoted to the prediction of transient responses under experimentally measured impact loading. Moreover, although classical laminated plate theory has been successfully applied to the static analysis of FDM structures, its capability to predict transient dynamic behaviour using experimentally identified modal damping has not yet been comprehensively validated. Consequently, a reliable and experimentally verified methodology for predicting the transient response of 3D-printed composite beams subjected to impulsive excitation is still lacking.

To address the above research gap, this study develops a hybrid experimental-numerical methodology for predicting the transient response of unidirectional 3D-printed polymer cantilever beams. Modal properties, such as resonant frequencies, mode shapes, and damping ratios, are identified through experimental modal analysis using a roving hammer technique and the circle-fit method. Transient response is then evaluated under hammer impact excitation applied at the beam mid-span point, with the response measured at the beam tip. An advancement in dynamic testing is achieved through the use of an ultra-light accelerometer with a mass of 0.2 [g]. A finite element model based on classical laminated plate theory is used to compute the beam’s mode shapes. These are combined with experimentally obtained modal damping values and the recorded impact force within a modal superposition framework to numerically predict the transient response. Modal participation factors and effective mass are calculated to establish the number of mode shapes required for accurate response prediction. By integrating experimental data with an efficient theoretical model, the proposed approach enables accurate prediction of the dynamic behaviour of layered, anisotropic FDM structures, thereby substantially increasing its engineering relevance. The presented research contributes to extending CLPT-based modelling toward the transient dynamic analysis of additively manufactured composite structures.

The main innovations of this work are as follows: (i) the development of a hybrid experimental-numerical framework that combines experimental modal analysis, measured impact loading, and CLPT-based finite element modelling for transient response prediction; (ii) the direct incorporation of experimentally identified modal damping ratios into the modal-superposition analysis; (iii) the assessment of the influence of the number of included vibration modes on transient response accuracy; and (iv) the experimental validation of the proposed methodology for a 3D-printed short carbon fibre-reinforced polyamide cantilever beam.

## 2. Materials and Methods

### 2.1. Material Description

**Figure 1:**
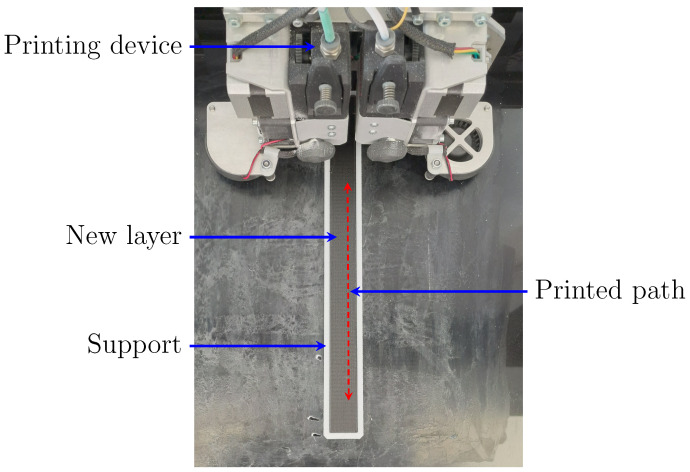
Additive manufacturing process of a unidirectional ($\varphi =0^{\circ }$) beam.

**Figure 2:**
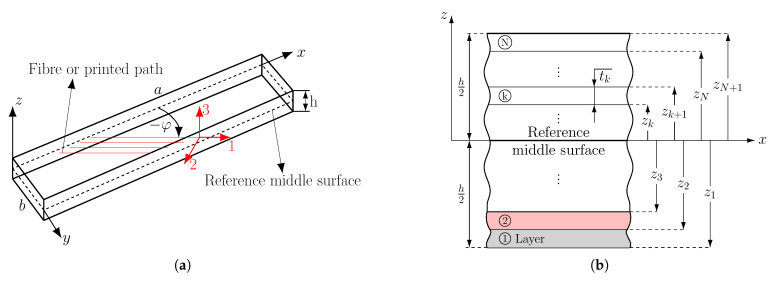
FDM-printed beam geometry and fibre/print path orientation: (**a**) 3-dimensional representation; (**b**) 2-dimensional representation of a beam cross section for the CLPT.

A unidirectional beam specimen was manufactured using fused deposition modelling (FDM). A polyamide CF-PA-12 filament with short carbon fibres was used to print the beam specimen. The filament was manufactured by IEMAI, Dongguan, China, and had a diameter of 1.75 [mm], a fibre content of 15%, and a mass density of $\rho =1070\;[\mathrm{kg}/m^{3}]$. Printing was performed using an industrial Omni 500LITE printer (Poznan, Poland). The Simplify3D slicer (version 4.1.2) was used with the printing parameters given in Table 1. In addition, the filament was dried at a temperature of 60 [°C] for 4 h before printing (with the WANHAO BOX 2 filament dryer, Jinhua, China). After the printing process, the beam was annealed in a conventional oven at a temperature of 80 [°C] for 6 h. Assuming an analogy to laminated composites, the fibre orientation of an individual layer corresponds to the filament printing path direction of the beam layer (see Figure 1). The geometric characteristics of the beam are shown in Figure 2. One beam specimen was considered in the present investigation. This choice was justified by the fact that the repeatability of the employed FDM manufacturing process and the experimental modal testing methodology had previously been demonstrated by the authors [32] using multiple nominally identical specimens manufactured with the same material, printer, and processing parameters as those adopted in the present work. The statistical analysis presented in that study demonstrated good repeatability of the identified dynamic characteristics, indicating that the adopted manufacturing process produced consistent specimens despite the inherent variability associated with FDM processes, such as interlayer bonding quality, porosity, and local filament orientation. Therefore, a single representative specimen was considered sufficient for validating the proposed transient-response prediction methodology.

### 2.2. Methodology

The methodology used in this study combines numerical modelling with experimental testing to predict the transient response of a 3D-printed composite cantilever beam subjected to impact loading. The main goal is to reproduce the time-dependent acceleration response of the beam using a realistic representation of the applied force.

**Figure 3:**
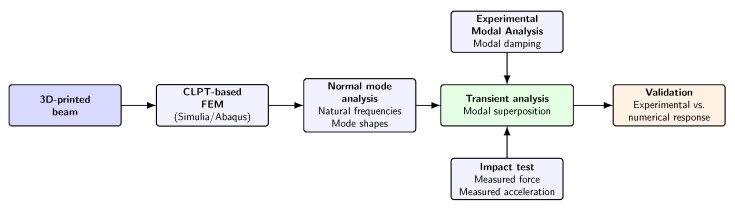
Flowchart of the proposed methodology.

The approach follows a hybrid experimental-numerical framework. First, a finite element model (FEM) of the beam is developed based on classical laminated plate theory (CLPT). A normal mode analysis is then carried out to identify the fundamental dynamic characteristics of the structure and to provide the basis for the transient response analysis. Next, experimental investigations are performed in two stages. Experimental modal analysis (EMA) is used to characterise the dynamic behaviour of the beam, particularly in terms of modal damping ratios. This is followed by an impact test, during which both the excitation force and the corresponding acceleration response are recorded. The measured force signal is used as input for the numerical model, while the experimental response serves as a reference for validation against a numerical prediction. Finally, a transient analysis is conducted using a modal superposition solution approach. Experimentally identified modal damping ratios are incorporated into the numerical model to accurately reproduce transient energy dissipation and vibration decay observed in the physical structure. For this purpose, the measured excitation force is used to compute the time-dependent response of the structure. This combined procedure allows for a direct comparison between numerical predictions and experimental results, providing a reliable and physically consistent framework for analysing the transient behaviour of additively manufactured composite structures.A schematic view of the whole procedure is shown on Figure 3.

### 2.3. Theoretical Background

A concise theoretical overview of the CLPT, numerical model, experimental modal analysis, and transient response analysis is provided to outline their fundamental features and highlight their applicability to the analysis of 3D-printed beams. Only the main aspects of the above methods are presented in this work due to the need for brevity and the fact that they are already well established. The reader may explore them in more detail using the references provided in the description of each method.

#### 2.3.1. Classical Laminated Plate Theory (CLPT) and Numerical Model

The main goal of CLPT is to predict the overall mechanical behaviour of a composite laminated beam by considering the properties of its individual layers, along with their stacking sequence and fibre orientations. The theory is based on the assumption that deformations remain small, which allows the material to be treated as linearly elastic. It also assumes that any line originally straight and perpendicular to the reference middle surface stays straight and normal to that surface after deformation [33]. In addition, CLPT simplifies the analysis by reducing a three-dimensional problem to an equivalent two-dimensional representation of a deformable body (see Figure 2). Under these assumptions, the displacement field in CLPT can be expressed as follows:

(1) $$u=u_{0}-z\alpha ,\;v=v_{0}-z\beta ,\;w=w_{0}$$

where $\left(u_{0},v_{0},w_{0}\right)$ are the displacement components along the (x,y,z) coordinate directions of a point on a reference middle surface (z=0), respectively; $\alpha =-\frac{\partial w_{0}}{\partial x}$, $\beta =-\frac{\partial w_{0}}{\partial y}$.

**Figure 4:**
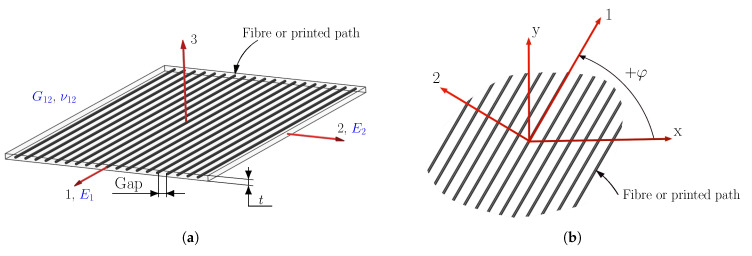
Single lamina configuration: (**a**) principal lamina coordinates; (**b**) off-axis configuration.

Each unidirectional lamina (layer) of the beam has a constant thickness *t* [mm] and is considered orthotropic under the plane stress state. For a layer *k* oriented in the 1–2 plane (along lamina principal coordinates 1–2), as shown in Figure 4a, the stress-strain relationship is given by the following:

(2) $${\left\{\begin{array}{c} \sigma_{1}\\ \sigma_{2}\\ \tau_{12} \end{array}\right\}}_{k}={\left[\begin{array}{ccc} Q_{11} & Q_{12} & 0\\ Q_{12} & Q_{22} & 0\\ 0 & 0 & Q_{66} \end{array}\right]}_{k}{\left\{\begin{array}{c} \varepsilon_{1}\\ \varepsilon_{2}\\ \gamma_{12} \end{array}\right\}}_{k}$$

where $\sigma_{1}$, $\sigma_{2}$, $\tau_{12}$ and $\varepsilon_{1},\varepsilon_{2},\gamma_{12}$ are in-plane stresses and strains, respectively; $Q_{ij}$ are reduced stiffnesses for the plane 1–2 (with the conventional Voigt notation adopted throughout the text). The $Q_{ij}$ are expressed in terms of engineering constants as follows:

(3) $$Q_{11}=\frac{E_{1}}{1-\nu_{12}\nu_{21}};\;Q_{22}=\frac{E_{2}}{1-\nu_{12}\nu_{21}};\;Q_{12}=\frac{\nu_{12}E_{2}}{1-\nu_{12}\nu_{21}};\;Q_{66}=G_{12};\;\frac{\nu_{12}}{E_{1}}=\frac{\nu_{21}}{E_{2}}$$

where $E_{1}$ and $E_{2}$ are extensional moduli in the 1- and 2-directions; $G_{12}$ is the shear modulus in the 1–2 plane; and $\nu_{ij}$ is Poisson’s ratio. For a single lamina oriented at angle (φ) to the *x* axis, as given in Figure 4b, a transformation of the stress-strain relations to the global coordinate system (x,y,z) must be applied. The stress-strain relations for a layer in the *x*–*y* plane become as follows:

(4) $${\left\{\begin{array}{c} \sigma_{x}\\ \sigma_{y}\\ \tau_{xy} \end{array}\right\}}_{k}={\left[\begin{array}{ccc} {\bar{Q}}_{11} & {\bar{Q}}_{12} & {\bar{Q}}_{16}\\ {\bar{Q}}_{12} & {\bar{Q}}_{22} & {\bar{Q}}_{26}\\ {\bar{Q}}_{16} & {\bar{Q}}_{26} & {\bar{Q}}_{66} \end{array}\right]}_{k}{\left\{\begin{array}{c} \varepsilon_{x}\\ \varepsilon_{y}\\ \gamma_{xy} \end{array}\right\}}_{k}$$

where ${\bar{Q}}_{ij}$ are the transformed reduced stiffnesses. For small strains (linear elasticity) and in the context of CLPT, there are only 3 non-zero strains of the reference middle surface, defined as (in short notation):

(5) $$\left\{\begin{array}{c} \varepsilon_{x}\\ \varepsilon_{y}\\ \gamma_{xy} \end{array}\right\}=\left\{\begin{array}{c} \varepsilon_{x}^{0}\\ \varepsilon_{y}^{0}\\ \gamma_{xy}^{0} \end{array}\right\}+z\left\{\begin{array}{c} \kappa_{x}\\ \kappa_{y}\\ \kappa_{xy} \end{array}\right\}$$

where $\varepsilon_{x}^{0}$, $\varepsilon_{y}^{0}$, $\gamma_{xy}^{0}$ are middle surface strains; $\kappa_{x}$, $\kappa_{y}$ are bending curvatures about the y- and x-axes, respectively; $\kappa_{xy}$ is a twisting curvature. The strains of an individual lamina can now be expressed in terms of the middle surface strains (Equation (5)) as follows:

(6) $${\left\{\begin{array}{c} \sigma_{x}\\ \sigma_{y}\\ \tau_{xy} \end{array}\right\}}_{k}={\left[\begin{array}{ccc} {\bar{Q}}_{11} & {\bar{Q}}_{12} & {\bar{Q}}_{16}\\ {\bar{Q}}_{12} & {\bar{Q}}_{22} & {\bar{Q}}_{26}\\ {\bar{Q}}_{16} & {\bar{Q}}_{26} & {\bar{Q}}_{66} \end{array}\right]}_{k}\left[\left\{\begin{array}{c} \varepsilon_{x}^{0}\\ \varepsilon_{y}^{0}\\ \gamma_{xy}^{0} \end{array}\right\}+z\left\{\begin{array}{c} \kappa_{x}\\ \kappa_{y}\\ \kappa_{xy} \end{array}\right\}\right]$$

In common structural applications, composite beams are constructed by stacking multiple unidirectional layers oriented at different angles (φ) to create a multi-layered structure. The constitutive equations of the multi-layered beam (or laminate) can be expressed in a contracted form as follows:

(7) $$\begin{array}{cc} \left\{N\right\} & =\left[A\right]\left\{\varepsilon^{0}\right\}+\left[B\right]\left\{\kappa \right\}\\ \left\{M\right\} & =\left[B\right]\left\{\varepsilon^{0}\right\}+\left[D\right]\left\{\kappa \right\} \end{array}$$

where {N} and {M} are the in-plane force and moment resultants, respectively, which are obtained by integrating the stresses in each layer through the beam thickness *h*; [A], [B] and [D] are 3×3 matrices of extensional stiffnesses, bending-extensional coupling stiffnesses, and bending stiffnesses, respectively. The elements of the matrices [A], [B] and [D] are given as follows:

(8) $$A_{ij}=\sum_{k=1}^{N}{\left({\bar{Q}}_{ij}\right)}_{k}\left(z_{k}-z_{k-1}\right);B_{ij}=\frac{1}{2}\sum_{k=1}^{N}{\left({\bar{Q}}_{ij}\right)}_{k}\left(z_{k}^{2}-z_{k-1}^{2}\right),D_{ij}=\frac{1}{3}\sum_{k=1}^{N}{\left({\bar{Q}}_{ij}\right)}_{k}\left(z_{k}^{3}-z_{k-1}^{3}\right)$$

where i,j=1,2,6; *N* is the total number of layers; $z_{k}$ and $z_{k-1}$ are the distances from the middle surface to the bottom and top of the $k^{\mathrm{th}}$ layer, respectively (see Figure 2b). The $A_{ij}$, $B_{ij}$ and $D_{ij}$ represent the equivalent stiffnesses of the multi-layered beam and allow the reduction of the 3-dimensional problem shown in Figure 2a to the 2-dimensional reference middle surface deformation. The stiffnesses depend on the layer stacking sequence, the fibre orientation angle (or printed path) (φ), four engineering constants, and the layer distance from the middle surface.

#### 2.3.2. Numerical Model

The scope of the current study requires the numerical model of the beam to undergo normal mode analysis to extract numerical eigenfrequencies and eigenvectors (mode shapes). The eigenvalue problem for undamped free vibrations is represented as follows:

(9) $$\left(K-\omega_{n}^{2}M\right)\phi_{n}=0$$

where K and M are the stiffness and mass matrices of the beam, respectively; $\phi_{n}$ are eigenvectors (mode shapes) corresponding to the eigenvalues (angular frequencies) $\omega_{n}=2\pi f_{n}^{\mathrm{FEM}}$ [rad/s], in which $f_{n}^{\mathrm{FEM}}$ are eigenfrequencies (cyclic frequencies [Hz]), and n=1,2,…,L. The normal mode analysis is conducted in order to solve Equation (9) and extract the eigenvalues and corresponding eigenvectors of the beam. The *n* eigenvalues and *n* natural modes form two matrices: a diagonal matrix called the spectral matrix $\Omega^{2}$ containing eigenvalues $\omega_{n}^{2}$, and a square matrix called the modal matrix $\Phi =\left[\phi_{1}\;\phi_{2}\;\phi_{3}\;\ldots \;\phi_{n}\ldots \;\phi_{L}\right]$, each column of which is a mode shape. The determination of the spectral and modal matrices is required in order to proceed to a transient response analysis.

**Figure 5:**
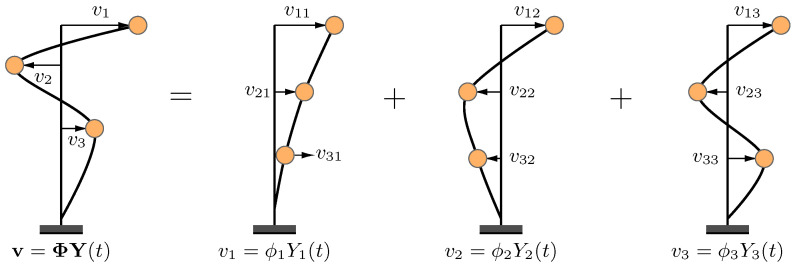
Schematic representation of the modal superposition approach for a multi-degree-of-freedom cantilever beam.

Transient analysis gives the response of the model as a function of time based on a given time-dependent loading [34]. Usually, transient analysis focuses on the structural response over the short time interval during which the applied force acts. This is called the force impulse duration. The current investigation considers a linear procedure used to evaluate transient linear dynamic problems through modal superposition. In this approach, the displacement field is represented as a linear combination of the system’s mode shapes and modal coordinates. Consequently, the governing partial differential equations of motion are transformed into a set of uncoupled single-degree-of-freedom equations expressed in modal coordinates. A graphical representation of the method is shown in Figure 5 and briefly described in the following. The dynamic equation of motion for multi-degree-of-freedom systems is expressed as follows:

(10) $$M\ddot{v}+C\dot{v}+Kv=P\left(t\right)$$

where M and K are defined as in Equation (9); C is the damping matrix; v is the displacement vector; and P(t) is the load vector.

By using a modal matrix of the system (Φ), the displacement vector v can be expressed in terms of modal coordinates (Y):

(11) $$v\left(t\right)=\sum_{n=1}^{L}\phi_{n}Y_{n}\left(t\right)=\Phi Y\left(t\right)$$

where *L* is the total number of modes included in the expansion. Substitution of Equation (11) (modal superposition) into Equation (10) yields uncoupled equations of motion in modal space for each mode *n*:

(12) $${\ddot{Y}}_{n}\left(t\right)+2\zeta_{n}\omega_{n}{\dot{Y}}_{n}\left(t\right)+\omega_{n}^{2}Y_{n}\left(t\right)=\frac{\bar{P_{n}}\left(t\right)}{\bar{M_{n}}}$$

where $\omega_{n}$ and $\zeta_{n}$ are the angular frequency and modal damping ratio of the mode *n*, respectively, $\bar{P_{n}}\left(t\right)=\phi_{n}^{T}P\left(t\right)$ and $\bar{M_{n}}=\phi_{n}^{T}M\phi_{n}$. Equation (12) must be solved for $Y_{n}\left(t\right)$ for each mode *n*. The modal superposition approach relies on the undamped modes of the system and can only be performed following a normal mode analysis, which provides mode shapes and angular frequencies. Equation (12) is now nearly populated in terms of known parameters ($\omega_{n},\;\phi_{n}$). The damping ratios $\zeta_{n}$ and the force time history (P(t)) can be provided by performing experimental modal analysis (EMA) and an impact test, respectively.

As the only unknown variable in Equation (12) left is $Y_{n}$, the FEM solution can be used for a time-domain transient analysis.

#### 2.3.3. Finite Element Model Implementation

The finite element model of the cantilever beam was developed using Simulia/Abaqus 2026. The beam was modelled as a multilayer composite laminate based on classical laminated plate theory (CLPT). Each printed layer was represented as an individual orthotropic lamina with a thickness corresponding to a printing layer thickness of 0.2 [mm]. The laminate consisted of twenty unidirectional layers with a stacking sequence of ${\left[\varphi \right]}_{20}$, corresponding to the printing path orientation adopted during specimen fabrication.

The numerical model employed four-node, reduced-integration, quadrilateral shell elements (S4R) equally distributed along the beam’s length. Orthotropic linear-elastic material behaviour was assumed for each lamina. The engineering constants used in the analysis were $E_{1}=8.05$ [GPa], $E_{2}=1.81$ [GPa], $G_{12}=0.82$ [GPa], and $\nu_{12}=0.48$. These values of the printed CF-PA-12 lamina were previously identified by the authors using an inverse material identification methodology based on experimental modal analysis and finite element model updating, as described in [32,35]. The identified engineering constants correspond to the same filament material, printer, and manufacturing procedure employed in the present study and were therefore adopted herein without further modification.

**Figure 6:**
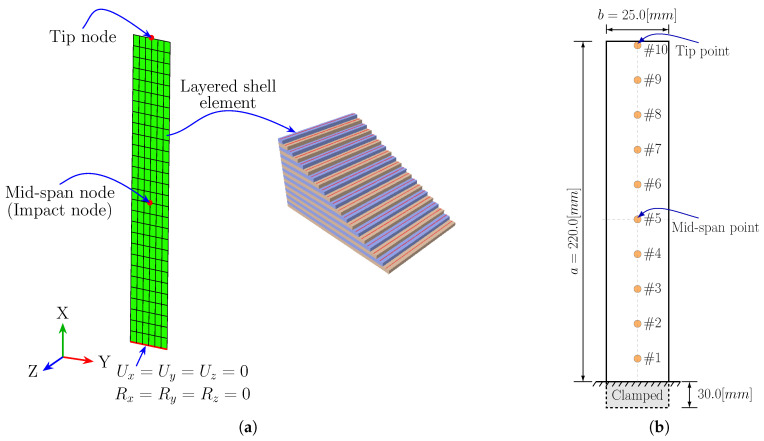
3D-printed cantilever beam: (**a**) FEM model; (**b**) beam geometry and measurements points.

The beam was modelled using the geometric dimensions shown in Figure 4a. The clamped boundary condition was implemented by fully constraining all translational and rotational degrees of freedom along the bottom edge of the beam (all nodes along the bottom edge—see Figure 6a), thereby reproducing the experimental cantilever support conditions.

The numerical analysis was performed in two stages. First, a linear perturbation *Frequency* analysis was carried out using the Lanczos eigensolver to extract the natural frequencies and corresponding mode shapes within the frequency range of 0–1800 Hz. Subsequently, a *Modal dynamics* analysis based on modal superposition was performed to predict the transient response. The experimentally measured impact force was applied as a time-dependent concentrated load at the beam mid-span node, while the experimentally identified modal damping ratios were introduced using the *Direct modal damping* option available in Abaqus. The solution stability was controlled by setting an appropriate time increment expressed as follows: $\Delta t\approx 1/\left(f_{L}^{FEM}\cdot 10\right)$, where $f_{L}^{FEM}$ is the largest cyclic frequency considered in the analysis. The *Modal dynamics* analysis is unconditionally stable, so that larger steps are possible as long as they represent the excitation amplitude and response frequency accurately.

The sufficient number of modes used for the transient analysis was controlled by reviewing two indicators: the modal participation factor and modal effective mass.

### 2.4. Experimental Modal Analysis

**Figure 7:**
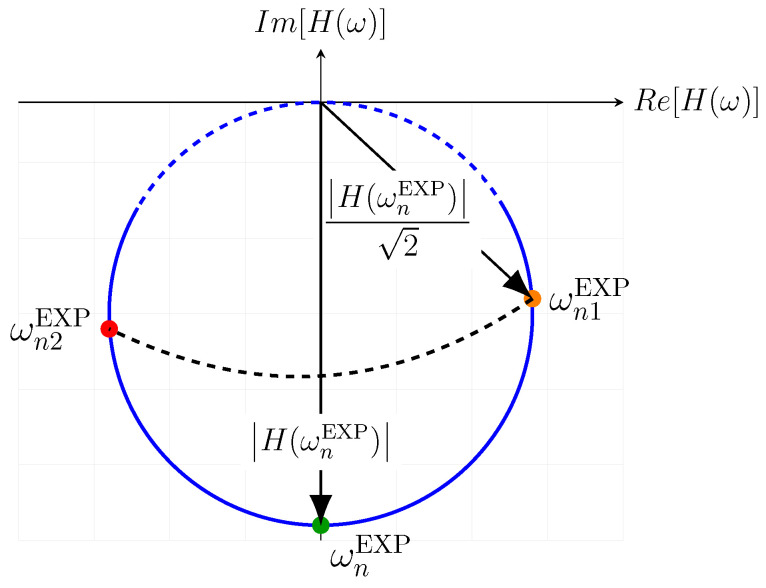
Schematic representation of a circle fit using a Nyquist plot—$\left|H(\omega_{n}^{EXP})\right|$ is the magnitude of FRF response at $\omega_{n}$.

Experimental modal analysis (EMA) is an established technique for determining the dynamic parameters of structures, such as resonant frequencies, damping ratios, and mode shapes [36]. It involves exciting the structure using a known input (e.g., an impact hammer or shaker) and measuring its response with sensors like accelerometers. By analysing the relationship between the input force and output response, frequency response functions (FRFs) are obtained as follows:

(13) $$H\left(\omega \right)=\frac{X(\omega )}{F(\omega )}$$

where H(ω) is a matrix containing the frequency response functions (FRFs), X(ω) is a vector of measured responses, F(ω) is a vector of applied forces, and ω [rad/s] is the angular frequency. The Nyquist plot (Re[H(ω)] vs. the imaginary part Im[H(ω)]) of the FRF is used for a least-squares fit of a circle near the $n^{th}$ resonance, followed by modal damping estimation as follows:

(14) $$\zeta_{n}=\frac{\omega_{n2}^{\mathrm{EXP}}-\omega_{n1}^{\mathrm{EXP}}}{2\omega_{n}^{\mathrm{EXP}}}=\frac{f_{n2}^{\mathrm{EXP}}-f_{n1}^{\mathrm{EXP}}}{2f_{n}^{\mathrm{EXP}}}$$

where $\omega_{n}^{\mathrm{EXP}}$ is the $n^{th}$ experimental resonant angular frequency; $\omega_{n1}^{\mathrm{EXP}}<\omega_{n2}^{\mathrm{EXP}}$ are angular frequencies associated with the Nyquist plot, as schematically shown in Figure 7; and $f_{n}^{\mathrm{EXP}}$, $f_{n1}^{\mathrm{EXP}}$, and $f_{n2}^{\mathrm{EXP}}$ are the corresponding experimental cyclic frequencies. The circle-fit method is a well-established frequency-domain identification technique that provides robust damping estimates, particularly for lightly damped structures, since it utilises the complex-valued FRF rather than only its magnitude. It is also less sensitive to frequency resolution and measurement noise [36].

Modal analysis techniques are classified based on the number of excitation inputs and measured responses. In SISO (single-input single-output), a single excitation and response are used, while SIMO (single-input multiple-output) employs one input with multiple response measurements. In contrast, MISO (multiple-input single-output) involves multiple excitations with a single response, while MIMO (multiple-input multiple-output) involves multiple simultaneous inputs and outputs. The choice of method is usually determined by the specific case and the complexity of the structure under investigation.

### 2.5. Experimental Setup

The study presents two experimental investigations conducted to analyse the transient response of a 3D-printed cantilever beam. First, the beam specimen was characterised through EMA, with the primary parameter of interest being the modal damping ratio associated with a selected mode shape. Additionally, mode shapes and resonant frequencies were extracted for validation against numerical results. Subsequently, an impact test was conducted in which the cantilever beam was excited using a modal hammer applied at the mid-span point, while the tip acceleration response was measured.

#### 2.5.1. Experimental Modal Analysis—Setup

**Figure 8:**
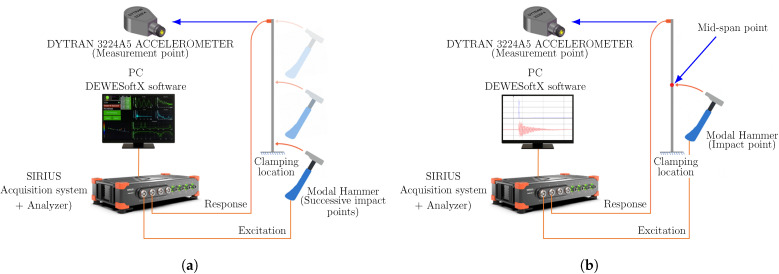
Experimental setups: (**a**) experimental modal analysis; (**b**) transient impact test.

The SISO-EMA method was employed, where SISO means single-input-single-output. The tests were conducted using a roving hammer approach, in which multiple excitation locations were defined while the response was measured at a fixed point (see Figure 8a). This configuration is well suited for medium-scale structures, providing an efficient means of obtaining high-quality modal data with minimal instrumentation. The beam was clamped with a heavy-duty metal fixture (a metal vice). For each impact, the excitation force and corresponding acceleration response were recorded and transformed into the frequency domain to compute the frequency response function (FRF). Repeating the excitation over a predefined grid yielded a set of FRFs describing the dynamic behaviour of the system. Data acquisition was performed using a DEWESoft SIRIUSi USB-powered 8-channel analyser (Trbovlje, Slovenia) within a frequency range of 0–1800 [Hz]. The acquired data were post-processed using DEWESoftX software (version 2023.4). The beam was excited using a modal hammer (Dytran 5800B3, HBK, Darmstadt, Germany) equipped with a plastic tip and force transducer having a sensitivity of 11.41 [mV/N]. The response was measured using a novel ultra-light accelerometer (Dytran 3224A5, HBK, Darmstadt, Germany), with a mass of 0.2 [g] and a sensitivity of 10.09 [mV/G], where G denotes gravitational acceleration. The accelerometer was mounted at the tip of the beam using wax adhesive. Ten evenly spaced excitation points were defined along the beam axis (see Figure 6b). At each excitation point, four hammer impacts were performed and averaged to improve the signal-to-noise ratio and reduce the influence of random variations in the impact force and contact conditions. The use of multiple impacts is standard practice in experimental modal analysis to improve the repeatability and reliability of the measured FRFs. Four impacts were considered sufficient to obtain stable and repeatable measurements while keeping the experimental time within practical limits. In addition, a double-hit protection feature was enabled to prevent multiple impacts at each excitation point. After completing all measurements, the modal parameters were extracted using the *Modal analysis* module.

#### 2.5.2. Transient Test—Setup

After completing the EMA, an impact test was carried out by hitting the beam at the mid-span point. During the impact, both the force time history and acceleration response at the tip point were recorded. The impact force was measured using the modal hammer (Dytran 5800B3), whereas the acceleration response was measured with the accelerometer (Dytran 3224A5). The same data acquisition system as in the EMA tests was used. A sampling increment of 0.0001 [s] was applied. The experimental setup of the impact test is shown in Figure 8b. The measured force signal was used as input for the numerical *Modal dynamics* analysis, while the tip acceleration served to validate the numerical results.

## 3. Results

The investigated 3D-printed composite beam consisted of 20 unidirectional layers with a printing path direction of φ=0 [deg]. Therefore, the total thickness of the beam was 4.0 [mm] with a layer stacking sequence of ${\left[0\right]}_{20}$. The beam dimensions were 220 [mm] × 25 [mm]. An additional 30 [mm] of the beam length was designed for the clamped zone to form the cantilever boundary condition (see Figure 6b).

**Figure 9:**
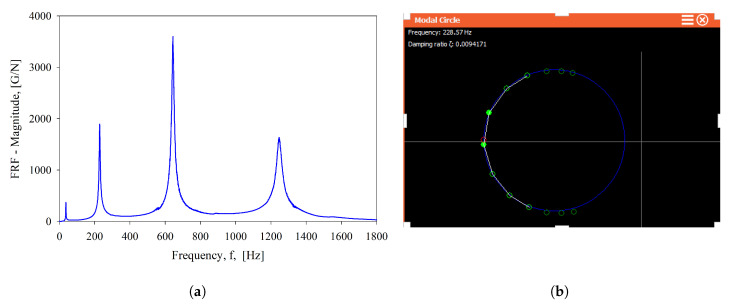
Experimental modal analysis results of the cantilever 3D-printed beam: (**a**) magnitude plot of the FRF; (**b**) example of the modal circle used for modal damping estimation.

**Figure 10:**
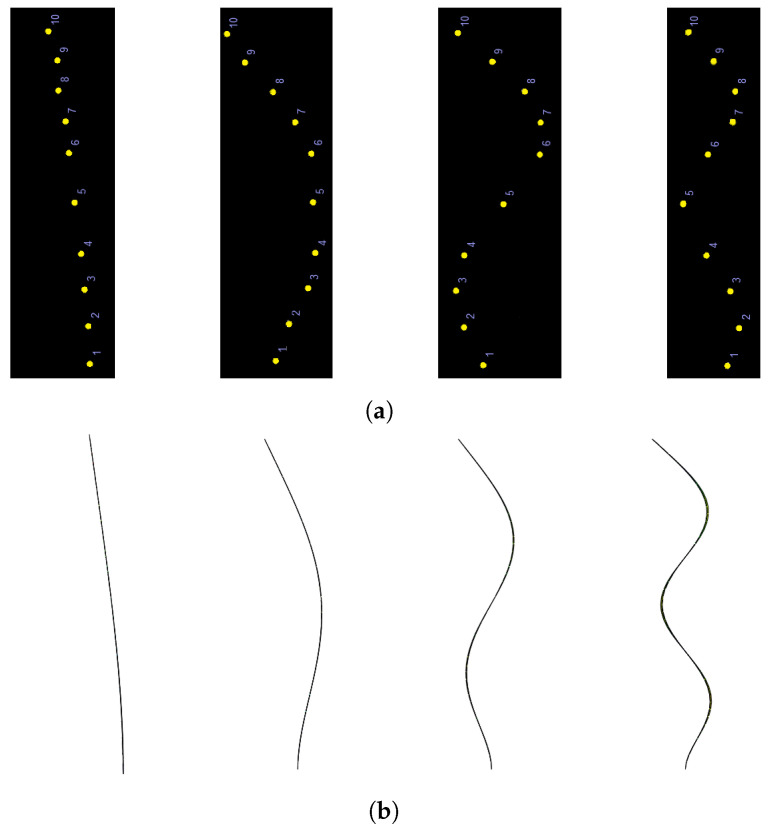
First four bending mode shapes extracted from: (**a**) experimental modal analysis; (**b**) FEM Frequency analysis.

The experimental modal analysis (EMA) of the cantilever beam enabled the identification of four bending modes of the beam. The magnitude plot of the obtained frequency response function (FRF) is shown in Figure 9a, with corresponding mode shapes presented in Figure 10a. The modal damping ratios were determined from the Nyquist plots using DEWESoftX software, as illustrated in Figure 9b. The extracted resonant frequencies and damping ratios are listed in Table 2. The modal damping ratios were identified using the Nyquist circle-fit method implemented in DEWESoftX. For each identified mode, a fitting bandwidth of 10 [Hz] and 13 fit points were used. These parameters were selected to include the complete resonance while excluding neighbouring modes, thereby ensuring a stable single-mode fit. The selected fitting parameters were kept identical for all repeated measurements to ensure consistency and reproducibility of the damping identification.

**Figure 11:**
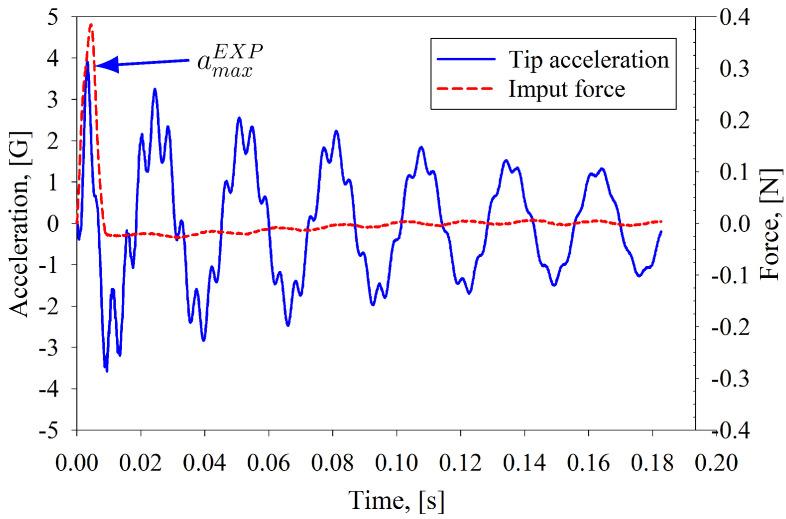
Experimental results—impulse force vs. acceleration response.

The transient impact test was conducted subsequently. The acceleration response at the tip point of the beam and the impact force history applied at the mid-span point were recorded. The measured force impulse and corresponding tip acceleration response are presented in Figure 11.

**Figure 12:**
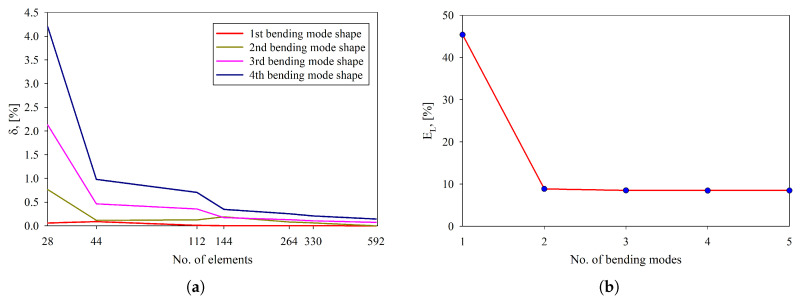
Sensitivity studies: (**a**) convergence solution study for the cantilever beam FEM model; (**b**) truncation study for transient analysis.

**Figure 13:**
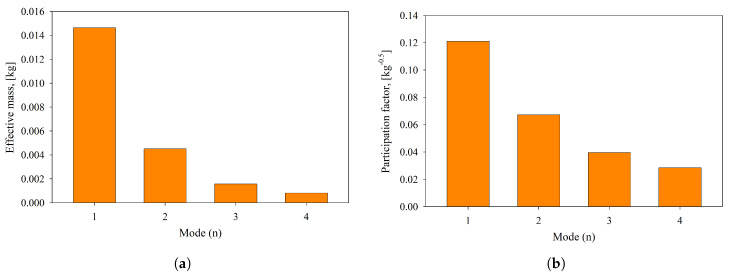
Numerical results: (**a**) variation of the effective mass; (**b**) variation of the participation factor.

Next, the FEM normal mode analysis was performed over the frequency range of 0–1800 [Hz] to extract bending mode shapes (see Figure 10b) and associated natural frequencies (see Table 2). The analysis was limited to the same four modes identified by EMA. Before proceeding with the FEM analysis, the convergence of the FEM solution (eigenfrequencies) was studied. The convergence measure was defined as the percentage difference in the angular frequency of each $n^{th}$ mode between successive analyses, as follows:

(15) $$\delta_{n}=\left|\frac{\omega_{n}^{i+1}-\omega_{n}^{i}}{\omega_{n}^{i}}\right|\cdot 100\%$$

where $\omega_{n}^{i}$ is the initial angular frequency associated with mode *n* for a coarse mesh, and $\omega_{n}^{i+1}$ is the angular frequency associated with mode *n* for a refined mesh. Satisfactory convergence was achieved with 264 uniformly distributed elements (see Figure 12a). The modal parameters extracted from the FEM model (eigenfrequencies and eigenvectors) are in good agreement with the experimental results, with deviations in cyclic frequencies below 5% (see Table 2). The normal mode analysis was verified in terms of the modal effective mass and modal participation factor. The values of these indicators for the response in the *z* direction are shown in Figure 13. The sum of the effective masses for the four bending modes is 0.0215 [kg], which is 90.1 [%] of the total mass of the model (0.0239 [kg]).

**Figure 14:**
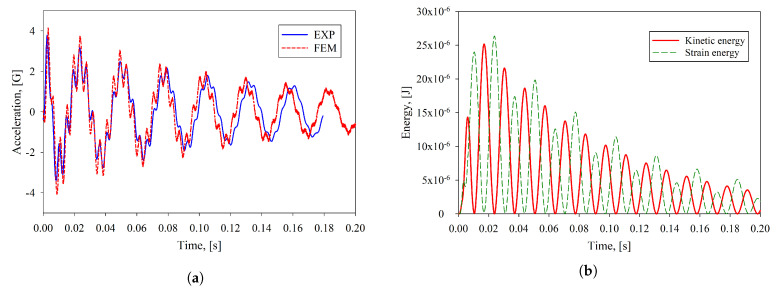
Experimental and numerical results: (**a**) comparison of the beam’s tip acceleration (FEM solution with 4 bending mode shapes included); (**b**) calculated kinetic and strain energies.

Next, the recorded force impulse was imported into the model as an external load applied at the mid-span node of the beam. The *Modal dynamics* analysis (transient response) was run using the modal damping ratios $\zeta_{n}$ listed in Table 2. The time increment was set as Δt=0.0001 [s]. A modal truncation convergence study was performed by comparing the calculated tip acceleration responses obtained with different numbers of bending modes against the experimentally measured maximum tip acceleration $a_{max}^{EXP}$ (see Figure 11). The relative response error was evaluated as follows:

(16) $$E_{L}=\left|\frac{{\left(a_{max}^{FEM}\right)}_{L}-a_{max}^{EXP}}{a_{max}^{EXP}}\right|\cdot 100\%$$

where ${\left(a_{max}^{FEM}\right)}_{L}$ is the calculated tip response obtained using *L* modes. The number of modes was considered sufficient when the change in the relative response error caused by adding further modes became negligible (see Figure 12b). The results confirmed that the response had converged well enough for the first four bending modes included. This is consistent with the modal effective mass criterion. As shown in Figure 14a, the tip point accelerations correspond very well, especially for the first few oscillations (up to 0.1 [s]). A small phase shift can be seen for the later oscillations, as a consequence of the discrepancies in the cyclic frequencies, as given in Table 2. However, the tip acceleration after mid-span impact showed a peak of 4.16 [G] (FEM) versus 3.84 [G] (experiment), differing by 8.46%. In addition, the FEM time history of the acceleration exhibits a vibration decay rate which matches well with the experimental results. This indicates that the damping ratios were extracted properly from EMA and that their implementation in the FEM is justified. As expected for a damped transient response, the kinetic and strain energies exhibit an alternating exchange throughout the vibration, reflecting the continuous conversion between the beam’s motion and elastic deformation. Simultaneously, the amplitudes of both energy components decrease with time due to the experimentally identified modal damping implemented in the numerical model. The observed energy decay is consistent with the reduction of the measured vibration amplitudes shown in Figure 14b, confirming that the proposed modelling approach realistically captures the dissipative behaviour of the structure. A quantitative comparison with experimentally determined energy quantities is beyond the scope of the present study and will be addressed in future work through dedicated experimental investigations.

## 4. Discussion

The results show that the proposed hybrid approach is able to reproduce the transient behaviour of the 3D-printed beam with good accuracy. The comparison of experimentally extracted cyclic frequencies with numerically calculated values indicates that the numerical model captures the frequencies well, with differences below 5%. The observed small deviations are most likely related to small imperfections in the clamping condition and the assumptions of the CLPT.

The close agreement between numerical and experimental responses confirms that the proposed modal framework captures the dominant transient dynamics of the structure, particularly during the initial phase after impact. The peak acceleration is slightly underestimated (by 8.46%), which can be attributed to minor experimental uncertainties, such as slight deviations in the impact location from the exact mid-span point and small discrepancies in the accelerometer placement. Despite this, the overall shape of the response and its decay are reproduced very well. This is a key observation, as it confirms that the damping has been accurately identified and correctly implemented in the numerical model. A small phase shift becomes noticeable at later times, which is most likely due to minor differences in natural frequencies that accumulate over the duration of the simulation. However, as the maximum tip acceleration occurs within the duration of the force impulse, it may be concluded that the main objective of the analysis was achieved. From the perspective of modal contribution, the results clearly show that the first and second bending modes dominate the response, while the first four bending modes together capture the majority of the system dynamics. This explains why a single-mode approximation is insufficient, whereas including four modes leads to a much closer agreement with the experimental data. At the same time, it highlights the efficiency of the modal superposition approach, as only a limited number of modes is required to accurately represent the transient response.

The present study is subject to several modelling and experimental limitations that should be considered when interpreting the results. The numerical model is based on classical laminated plate theory (CLPT), which assumes linear elastic material behaviour, small deformations, and neglects transverse shear deformation. These assumptions are generally appropriate for thin laminated structures subjected to low-amplitude vibrations, as in the present investigation. Nevertheless, they may become less accurate for thick laminates, highly nonlinear responses, or large deformation problems.

The numerical model also assumes ideal material properties and perfect layer geometry. In practice, FDM-manufactured composite structures inevitably contain process-induced imperfections, including small variations in filament dimensions, local porosity, imperfect interlayer bonding, residual stresses, and minor geometric deviations. Such imperfections can locally modify the stiffness, mass distribution, and damping characteristics of the beam, contributing to small differences between numerical predictions and experimental observations.

Experimental uncertainties should also be considered. The boundary condition provided by the mechanical clamp cannot be regarded as perfectly rigid, while slight variations in impact location, impact force, accelerometer positioning, sensor mounting, and measurement noise may additionally influence the measured dynamic response. Despite these unavoidable sources of uncertainty, the numerical model predicts the resonant frequencies with deviations below 5% and reproduces the transient acceleration response with good agreement, indicating that the adopted methodology provides a reliable representation of the dynamic behaviour of the investigated structure.

Overall, the results demonstrate that CLPT-based modelling combined with experimentally identified damping enables accurate prediction of transient response in FDM composite beams. The approach shows a good balance between accuracy and computational efficiency, making it suitable for engineering applications where dynamic loading needs to be assessed.

## 5. Conclusions

The current study presents a hybrid experimental-numerical methodology for predicting the transient response of a 3D-printed composite cantilever beam made from short carbon fibre-reinforced polyamide. The approach combines experimental modal analysis (EMA) with a CLPT-based finite element model and modal superposition. The findings of this study have several important implications:

1. The strong agreement between EMA and FEM confirms that classical laminated plate theory can successfully represent the anisotropic behaviour of 3D-printed composites.
2. Accurate transient predictions can be achieved using only a limited number of modes, making the approach computationally efficient.
3. Incorporating experimentally derived damping is essential for realistic transient response prediction, capturing vibration and energy decay phenomena.
4. The methodology is applicable to structures subjected to impact or transient loads, such as 3D-printed lightweight structural components.

The present research confirms that combining CLPT-based modelling with experimentally derived modal parameters is a reliable strategy for analysing the transient dynamics of FDM composite structures. The proposed methodology offers a practical tool for the design and assessment of additively manufactured components subjected to dynamic and impact loading.

Although the proposed methodology demonstrates good agreement with the experimental results, future work will focus on further improving its predictive capability by incorporating manufacturing-induced imperfections and experimentally quantifying energy dissipation. In particular, the proposed framework will be extended to 3D-printed plate-like composite structures, which exhibit more complex dynamic behaviour due to the presence of closely spaced and interacting vibration modes. Special attention will also be devoted to assessing the influence of modal damping identification techniques on the accuracy of transient response predictions.

## Figures and Tables

**Table 1:** Printing parameters.

Parameter	Value
Layer thickness	0.2 [mm]
Nozzle diameter	0.4 [mm]
Infill density	100.0 [%]
Infill pattern (Rectilinear)	0.0 [deg]
Nozzle temperature	300.0 °C
Bed temperature	100.0 °C
Chamber temperature	50.0 °C

**Table 2:** Comparison of experimental modal analysis and numerical Frequency analysis results.

Mode *n*	Type	$f_{n}^{\mathrm{EXP}}$ (Hz)	$f_{n}^{\mathrm{FEM}}$ (Hz)	Δ (%)	$\zeta_{n}$
1	Bending	35.52	37.30	4.8	0.0240
2	Bending	228.57	232.70	2.0	0.0094
3	Bending	644.08	648.20	0.6	0.0083
4	Bending	1244.47	1261.10	1.3	0.0018

## Data Availability

The original contributions presented in this study are included in the article. Further inquiries can be directed to the corresponding author.
